# Differences in the Ratios of General and Dental Specialists in Europe

**DOI:** 10.1016/j.identj.2023.12.004

**Published:** 2024-01-16

**Authors:** Javier Fernández-Serrano, Eugenia García-Espona, José Antonio Alarcón, Cristina García-Espona, Ignacio García-Espona

**Affiliations:** a Department of Stomatology, Section of Orthodontics, School of Dentistry, University of Granada, Granada, Spain; b President of the Spanish Association of Orthodontists (AESOR), Madrid, Spain

**Keywords:** Dental specialties, Orthodontics, Oral surgery, Europe

## Abstract

**Background:**

The European Economic Area (EEA) is composed of member states with a multitude of different regions. This study aimed to analyse the ratios of general dentists and dental specialists to the total population and the proportion of dental specialists to general dentists in 24 European countries and to explore specific intranational differences within 2 countries: France and Germany.

**Methods:**

Available official documents and webpages from the United Kingdom and 23 of the 30 countries comprising the EEA were analysed. Data were expressed as absolute values, ratios of general dentists and dental specialists in the total of population, and percentages of dental specialists/dentists. The Mann–Whitney *U* test was used to clarify the main ratios that distinguish France from Germany, and cluster analysis was employed to determine similar areas.

**Results:**

Significant differences were found between countries, with Ireland and Austria having the lowest ratio of dentists and Romania and Greece having the highest. The Czech Republic, the Netherlands, France, and Denmark had the lowest ratios of dental specialists to the total population. Lithuania, Sweden, and Germany had the highest number of dental specialists. Orthodontists were the most numerous specialists (5.0% of dentists), followed by oral surgeons (2.7%). In France, differences between departments were pronounced and associated with the presence of dental schools and per capita income. In Germany, only the correlation between per capita income and the density of oral surgeons was significant.

**Conclusions:**

Diverse ratios of general dentists and dental specialists to the total population and the proportion of dental specialists to general dentists were discovered within the examined countries, and their maximum values were 2.5, 5.7, and 4.1 times the minimum values, respectively. Differences were even found within the same country, as was the case in France and, to a lesser extent, in Germany.

## Introduction

The European Economic Area (EEA) consists of 30 member states with a multitude of different regions and economically and politically regulated territories. Historically, despite having common European legal guidelines, such as the 2005/36/CE and 2013/55/EU, each country has developed its own health system, with strong implications for dentistry. Significant differences exist in the ratio of dentists in the total population and number and types of officially recognised dental specialties between countries and even between regions of the same country.^1^, ^2^, ^3^, ^4^, ^5^, ^6^, ^7^, ^8^, ^9^, ^10^, ^11^, ^12^ With regard to funding, the public, private, and hybrid health care systems differ extensively, thus impacting the number and distribution of oral health care professionals in European countries. There are economic and salary discrepancies amongst European citizens, and in several countries, universal coverage for oral health care within the overall health care system is lacking. These variables affect the working conditions and numbers of dental professionals because in places where more patients seek dental services, more dental professionals are required to cover the higher demand.^13^

In this context, the training of future professionals is of special relevance. The annual number of students admitted to dental schools is controlled in Germany, Italy, France, Greece, Ireland, Poland, Sweden, and the UK but is less so in Romania, Portugal, and Spain. Consequently, there is a surplus of dentists in Romania, Portugal, and Spain (with a high number of private dental schools), and many newly graduated dentists in these countries are underemployed or unemployed. In the short term, overproduction can benefit other countries, such as the UK, which have trained insufficient numbers of their own nationals (resulting in a low ratio of dentists/population).^10^

This research analyses the ratios of general dentists and dental specialists to the total population and the proportion of dental specialists to general dentists in 24 European countries in order to test inequalities in the general and specialised dental services offered to patients. Moreover, it investigates specific internal differences between 2 countries—France and Germany—analysing the relationship between the density of professionals (general dentists, orthodontists, and oral surgeons) and the existence of dental schools and per capita income.

It is hoped that the results of this study will prove useful for the development and standardisation of oral health systems and for the training of oral health care professionals in European countries.

## Materials and methods

Available official documents and webpages, mainly linked to regulatory bodies, official colleges and councils, and dental institutions from 23 of the 30 countries comprising the EEA and the UK were collected and analysed to obtain reliable data regarding the number of general and specialised dentists. Additional documents, such as annual reports, were considered to extend and corroborate the publicly available information.^14^, ^15^, ^16^, ^17^, ^18^, ^19^, ^20^, ^21^, ^22^, ^23^, ^24^, ^25^, ^26^, ^27^, ^28^, ^29^, ^30^, ^31^, ^32^, ^33^, ^34^, ^35^, ^36^, ^37^ Eight countries from the EEA (Bulgaria, Croatia, Slovakia, Slovenia, Estonia, Hungary, Latvia, and Malta) were not included due to the lack of sufficient reliable information sources.

Data were collected as absolute values (total population, number of general dentists, number of dental specialists) and as ratios of general dentists/population, dental specialists/population, and percentages of dental specialists/dentists.

An internal analysis was conducted in France and Germany, 2 countries with a clear regional or federal structure and for whom reliable and updated data segregated by specific areas (ie, departments and federal state dental chambers in the cases of France and Germany, respectively) were available. At this level, we only considered 2 specialties, orthodontics and oral surgery, mainly because they were the most frequent dental specialties in Europe^12^ and the only common specialties within the entire territory of both countries. Other specialties, such as oral medicine in France or periodontics, public health, or conservative or preventive dentistry, which are only recognised in some federal state dental chambers in Germany (Westfalen-Lippe and Nord Rhein) were not considered.

To determine the main ratios that distinguish France from Germany, we used the Mann–Whitney *U* test, which assesses whether the difference between the medians of 2 non-Gaussian variables, once weighted (W), is sufficiently large to establish a significant difference between them. The variables considered for this objective in our study were as follows:

**Box 1:** Study Variables.

Abbreviation	Variables
*Pop*	Population by region
*Dent*	Total number of dentists by region
*Orth*	Total number of orthodontists by region
*OralSur*	Total number of oral surgeons by region
*Rdent*	Number of dentists per 100,000 population
*Prop*	Proportion of orthodontists relative to the total number of dentists
*Rorth*	Number of orthodontists per 100,000 population
*RoralSur*	Number of oral surgeons per 100,000 population

The statistical analysis included cluster analysis to determine similar groups (in this case, areas). Each area was represented as a vector whose components were the values of the variables *Rdent, Rorth, Prop*, and *RoralSur*. The distance between 2 areas was defined as the Euclidean distance between the 2 vectors that represent them. Based on this definition, cluster analysis allowed us to obtain and represent all areas, thus highlighting the groups and associations of similar areas. The resulting information was synthesised using a dendrogram, that is, a fork diagram where each fork groups areas at its lower level. The height of each fork indicates the maximum distance between the areas that form it.

Finally, the association between the presence of dental schools and per capita income with the number of professionals was analysed using the Spearman correlation analysis or the Mann–Whitney *U* test according to the variables to be related.

We used R (4.2.0) and IBM SPSS 29 for statistical calculations and graphs.

## Results

**Fig. 1:**
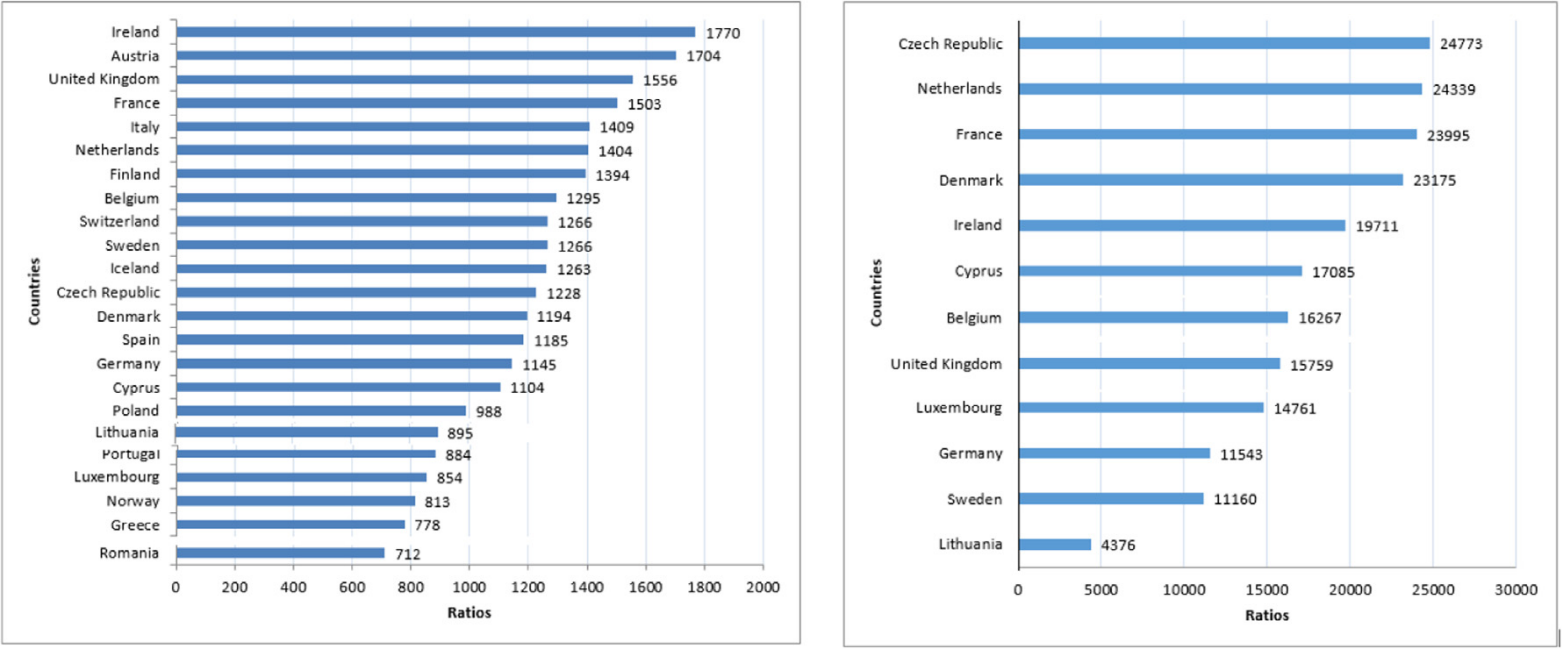
Ratios of dentists to the total population (left) and dental specialists to the total population (right). Reference year: 2022. Last access date: 5 May 2022.

Table 1 and Figure 1 show the total population, the number of general dentists, the number of dental specialists, the ratios between general dentists/population and dental specialists/population, and the percentage of dental specialists/general dentists in each analysed country. The reference years used for population, number of dentists, and dental specialists were 2020 through 2022, excluding Belgium (2019). For a total population of 494,703,850 in the analysed countries, there were 405,889 general dentists, which equates to 1 general dentist for every 1219 people. The ratios of general dentists/population varied from a low of 1/712 in Romania to a high of 1/1770 in Ireland. The ratio of dental specialists/population was 1/15,417, with the highest and lowest ratios belonging to Lithuania (1/4376) and the Czech Republic (1/24,773), respectively. Amongst all analysed countries with reliable data for dental specialists, the percentage of dental specialists/general dentists was 8.7%, ranging from a low of 5% in the Czech Republic to a high of 20.4% in Lithuania.

**Table 1:** Total population, number of general dentists, number of dental specialists (in total), ratios of general dentists and dental specialist to populations, and percentage of dental specialists/general dentists in the analysed European countries.

	Total population	General dentists	Ratio dentists/ population	Dental specialists	Ratio specialists/ population	% Dental specialists/ general dentists
Austria	8,932,664	5242	1/1704	*	*	*
Belgium	11,566,041	8930	1/1295	711	1/16,267	8.0%
Cyprus	956,800	866	1/1104	56	1/17,085	6.5%
Czech Republic	10,701,777	8713	1/1228	432	1/24,773	5.0%
Denmark	5,840,045	4890	1/1194	252	1/23,175	5.2%
Finland	5,533,793	3969	1/1394	-	-	-
France	65,627,454	43,646	1/1503	2735	1/23,995	6.3%
Germany	83,155,031	72,591	1/1145	7204	1/11,543	9.9%
Greece	10,682,547	13,737	1/778	-	-	-
Iceland	368,792	284	1/1263	-	-	-
Ireland	5,006,709	2828	1/1770	254	1/19,711	9.0%
Italy	59,257,566	42,044	1/1409	-	-	-
Lithuania	2,857,276	3194	1/895	653	1/4376	20.4%
Luxembourg	634,730	743	1/854	43	1/14,761	5.8%
Netherlands	17,475,415	12,448	1/1404	718	1/24,339	5.8%
Norway	5,391,369	6625	1/813	-	-	-
Poland	37,840,001	38,440	1/988	-	-	-
Portugal	10,291,027	11,640	1/884	-	-	-
Romania	19,186,201	26,931	1/712	-	-	-
Spain	47,326,687	39,919	1/1185	*	*	*
Sweden	10,379,295	8197	1/1266	930	1/11,160	11.3%
Switzerland	8,812,728	6958	1/1266	-	-	-
UK	67,025,542	43,054	1/1556	4253⁎⁎	1/15,759⁎⁎	9.9%⁎⁎
Total	494,703,850	405,889	1/1219	18,241	1/15,417	8.7%

⁎ As Austria and Spain did not recognise officially dental specialties at the beginning of this study, data about specialists were not available.

⁎⁎ The figure of 4253 dental specialists is the total number of names in the UK on all 13 specialist lists and not the number of specialists as some of them are named on more than one specialist list, which might affect the ratios specialists/population and dental specialists/general dentists. Reference years for population and number of dentists and specialists: 2020–2022 (Belgium: 2019). Last access date: 5 May 2022 (Lithuania and Switzerland: 31 October 2023).

Table 2 shows the number of dental specialists per country and the percentage of specific dental specialty/total general dentists. Countries without specific data are not listed. The specialty with the largest number of professionals in all analysed countries was orthodontics, with a total of 12,734 specialists, representing 5.0% of all dentists in those countries with that dental specialty. The lowest and highest percentages of orthodontists/total number of dentists were found in Portugal (0.6%) and Switzerland (8.1%), respectively. The dental specialty with the second largest number of professionals was oral surgery, with 5008 specialists (2.7% of all dentists in those countries with that dental specialty). Oral maxillofacial surgeons performing oral surgery were not counted (324) because the European Commission lists oral and maxillofacial surgery as a medical speciality rather than a dental one.

**Table 2:** Number of dental specialists per country and percentage of specific dental specialty/total general dentists.

	Orthodontics	Oral surgery	Others
Austria	⁎⁎	⁎⁎	⁎⁎
Belgium	517 (5.8%)	⁎⁎⁎	194 (2.2%)
Cyprus	38 (4.4%)	18 (2.1%)	0
Czech Republic	300 (3.4%)	92 (1.1%)	40 (0.5%)
Denmark	175 (4.1%)	77 (1.6%)	0
Finland	155 (3.9%)	-	-
France	2428 (5.6%)	210 (0.5%)	97 (0.2%)
Germany	3752 (4.3%)	3452 (4.8%)	0
Iceland	14 (4.7%)	-	-
Ireland	182 (5.4%)	72 (2.5%)	0
Italy	1202 (2.9%)	-	-
Luxembourg	34 (4.7%)	9 (1.2%)	0
Netherlands	361 (2.9%)	357* (2.9%)	0
Poland	1291 (3.4%)	-	-
Portugal	89 (0.6%)	-	-
Spain	⁎⁎	⁎⁎	⁎⁎
Sweden	273 (3.3%)	-	657 (8.0%)
Switzerland	562 (8.1%)	-	-
UK	1361 (3.2%)	721 (1.7%)	2171 (5.1%)
Total	12,734 (5.0%)	5008 (2.7%)	3,159

⁎ The Netherlands officially recognises maxillofacial surgery as a dental specialty instead of oral surgery, which is included in it.

⁎⁎ Countries without officially recognised dental specialties at the beginning of the study.

⁎⁎⁎ Belgium does not recognise oral surgery as a dental specialty. Therefore, oral maxillofacial surgeons (324 in 2021) performing oral surgery were not counted. Reference period for population and number of specialists: 2020–2022 (Belgium: 2019). Last access date: 5 May 2022 (Switzerland: 31 October 2023).

The lowest and highest percentages of oral surgeons were found in France (0.5%) and Germany (4.8%), respectively. Data on other dental specialties, such as periodontics and oral medicine, were available in only a few of the analysed countries.

Supplementary Tables 1 and 2 present an internal analysis of France and Germany, respectively. For each area, the total population and the number of dentists, orthodontists, and oral surgeons (*Dent, Orth, OralSur*), are shown in absolute values and as ratios per 100,000 population (*Rdent, Rorth, RoralSur*). The Mann–Whitney *U* test revealed significant differences between the areas of France and Germany, regarding *Rdent, Rorth*, and *RoralSur* (all *P* = .000; see Table 3). For all variables, the analysed indicators were higher in the German areas (federal state dental chambers) than in the French regions.

**Table 3:** Contrast between the areas of France and Germany (Mann–Whitney *U* test).

*Country*	*Rdent*	*Prop*	*Rorth*	*RoralSur*
France	58.90 ± 19.17	4.65 ± 1.72	2.90 ± 1.64	0.23 ± 0.31
Germany	88.11 ± 13.19	4.87 ± 0.74	4.30 ± 0.94	4.02 ± 1.07
	*U* = 135	*U* = 810	*U* = 350	*U* = 0
	*P = .000	*P = .710	*P = .000	*P = .000

Data are presented as means and standard deviations.

⁎ Mann–Whitney *U* test. *Rdent,* ratio of dentist per 100,000 population; *Prop*, proportion of orthodontists relative to total of dentists; *Rorth,* ratio of orthodontists per 100,000 population; *RoralSur,* ratio of oral surgeons per 100,000 population.

Supplementary Figures 1 and 2 display the geographic behaviour and stratification of the density of dentists and orthodontists based on the *Rdent* and *Rorth* variables in France and Germany, respectively, using a blue colour scale. For the purpose of simplification, only the number of orthodontists (the most frequently recognised dental specialty in Europe) is displayed. Paris and its metropolitan area and the areas located in the French periphery had higher *Rdent* and *Rorth* values; this difference was substantially more pronounced in the areas of southern France. In contrast, in Germany, there was no clearly defined geographical gradient.

The relationship between the density of different professionals (general dentists, orthodontists, and oral surgeons) with the presence of dental schools and per capita income in both countries was also analysed. In France, the Mann–Whitney *U* test showed significant differences amongst *Rdent, Rorth*, and *RoralSur* in terms of the existence of dental schools (*P* = .000), and the correlation in the different population areas amongst these 3 rates and per capita income was significant (*P* = .000; Spearman correlation). In Germany, only the correlation coefficient between per capita income and *RoralSur* was significant (*P* = .044; Spearman correlation).

**Fig. 2:**
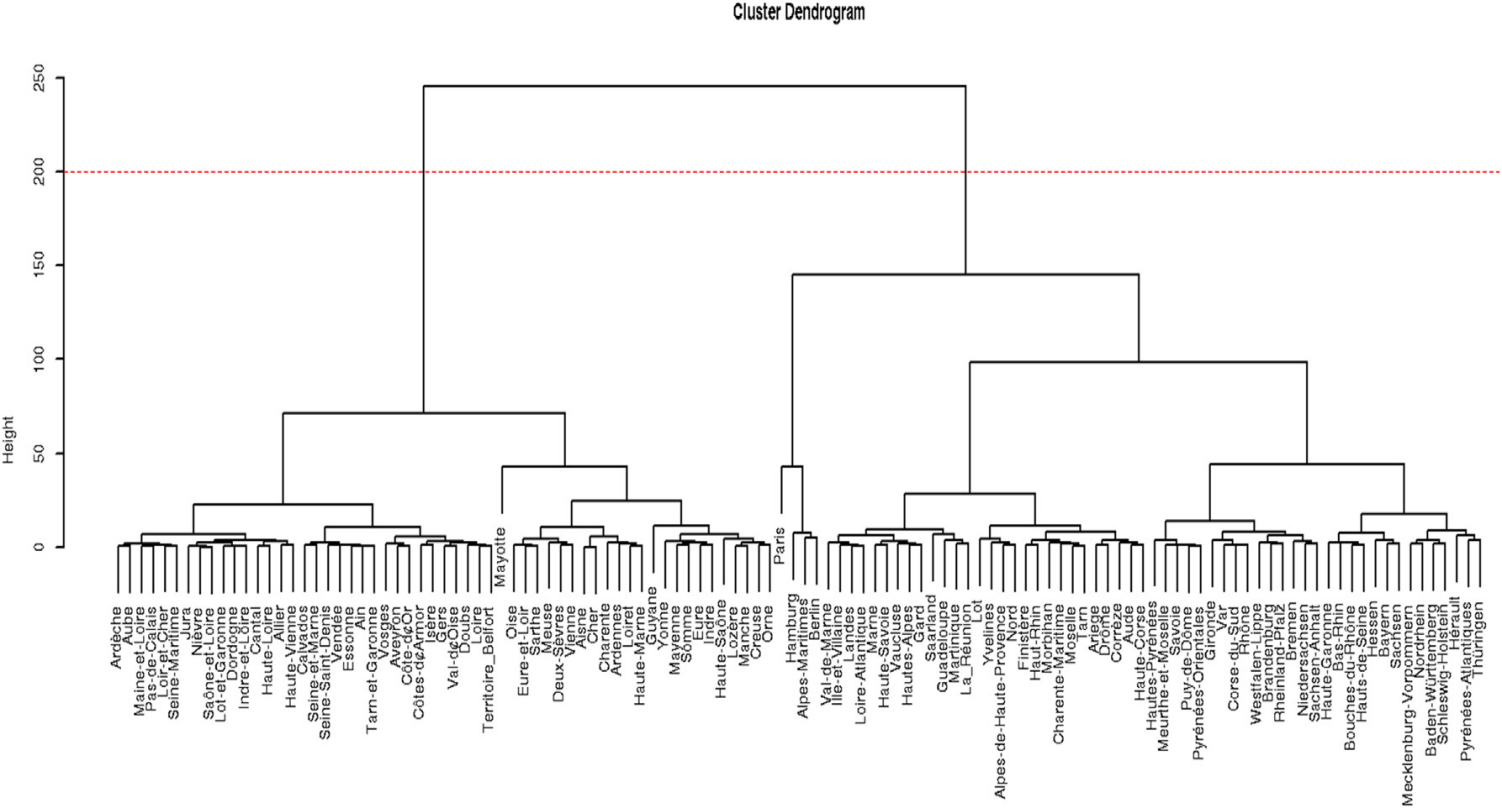
Cluster dendrogram of the analysed areas of France and Germany.

Figure 2 shows the dendrogram obtained by applying cluster analysis to all areas of France and Germany. Using a cutoff of 200 on the vertical axis, we obtained 2 large groups of areas that were clearly defined according to their dental similarity. All the areas of Germany were grouped in the same cluster, suggesting greater intranational homogeneity than France, with departments distributed in both clusters.

## Discussion

### EEA national differences

The EEA countries and the UK presented different data. The ratio of dentists/populations differed significantly amongst the analysed countries—1/712 in Romania to 1/1770 in Ireland—whilst the ratio for all analysed countries (1/1219) was well above the minimum ratio recommended by the World Health Organization (1/3500).^38^ These significant differences were also evident when all the specialised dentists were analysed. The ratio of dental specialists/population was the lowest in Lithuania, Sweden, and Germany and the highest in the Czech Republic, the Netherlands, France, and Denmark.

The total percentage of dental specialists/dentists in all analysed countries was 8.7%, oscillating between 5% in the Czech Republic to 20.4% in Lithuania, which is a statistical anomaly in the EEA context because it is 9 percentage points above the second country in this scale, Sweden, with 11.4%. Spain and Austria were excluded owing to the absence of officially recognised dental specialties at the beginning of this study (orthodontics was recognised as an official dental specialty in Austria on 27 February 2023), although this is mainly a legal problem and not a problem of having qualified dental specialists.

Several cofactors can influence the data concerning the number of dentists and specialists and their respective ratios, which can be conditioned by other characteristics such as the regional economic situation, associated health level, presence or absence of official studies related to dentistry, and population size and dispersion.

Although the reported data may be outdated due to the constant flux in the development of dental specialties in the European region, the current information provides indicative information for stakeholders, including decision-making bodies (eg, national dental councils) to make relevant comparisons and review local health care needs for the future. Additional relevant information can be found on the Council of Dental Chief Dental Officers (CECDO) webpage.^39^

Our analysis mainly focused on the 2 historical and most frequently officially recognised dental specialties—orthodontics and oral surgery—due to the fact that they approximately double the prevalence of other specialties.^12^ The overall proportion of orthodontists/dentists was 5.0%, varying from 0.6% in Portugal to 8.1% in Switzerland. The proportion of oral surgeons/dentists ranged from 0.5% in France to 4.8% in Germany, with an overall proportion of 2.7%. Orthodontists were more prevalent than oral surgeons in all countries where reliable data were obtained, with the greatest difference in the case of France (2428 orthodontists vs 210 oral surgeons). Other dental specialties were not evaluated as deeply as orthodontics and oral surgery because they are officially recognised in only a few countries, and in some countries these data are not made available to the general public.^40^

The case of the UK is striking, as it has 13 different officially recognised dental specialties. Orthodontics is the most common specialty in the country, followed by oral surgery, prosthodontics, and periodontics (as of 31 December 2022).

Our results demonstrate that ratios of general dentists and dental specialists to the total population and proportions of dental specialists to general dentists are diverse in the EEA and the UK, and their maximum values are 2.5, 5.7, and 4.1 times the minimum values in the analysed countries. Therefore, harmonisation at the European level to solve these clear differences is recommended. Improvements for this harmonisation could include different actions. We suggest several measurements, such as a more precisely defined European legal and administrative regulations concerning dentistry. Second, it is necessary to expand the knowledge of these obvious inter-European differences through dental regulatory bodies, official college councils, and scientific societies. Third, the European Commission should seek to improve the opportunities for dental professionals to move freely between EEA countries and enhance the current system for the recognition of academic and professional qualifications. Last but not least, there is need to create a more homogeneous common pattern for the distribution of dental faculties to train dental professionals in a more balanced way. Many other measurements could be considered to harmonise European dental services.

The main European guidelines concerning dental specialties (78/686/CEE, 78/687/CEE, 78/688/CEE, and more recently the 2005/36/CE and 2013/55/EU directives) explicitly recognise the first 2 professional dental specialisations—orthodontics and oral surgery—thereby empowering the European Commission to include new dental specialties common to at least two-fifths of the member states. Furthermore, establishing a minimum mandatory number of dental specialties for all EEA countries would be useful to enhance specialist dental harmonisation, thereby forcing Spain to officially recognise dental specialties.

### The cases of France and Germany: intranational differences

A deeper analysis was conducted on the internal situation in 2 countries with a clear administrative and professional division in the EEA: “departments” and “regions” in France and “federal state dental chambers” in Germany and for whom disaggregated and updated data were available. The number of areas was remarkably similar when the French departments were grouped into 18 regions, which is close to the 17 German federal state dental chambers. However, it should be noted that when dealing with ratios and proportions, the "population effect" as a mediating variable is eliminated.

The German areas showed significantly higher ratios of dentists and orthodontists or oral surgeons/dentists than the French regions. To explain this finding, we tested whether the density of dental professionals was related to the presence of dental schools or the per capita income. Nevertheless, future studies should consider a higher number of countries and additional cofactors that could explain the differences in the analysed variables, such as the associated level of health, volume and type of economic activity, university educational level in relation to health sciences, the political and administrative importance of the area at the national level, and public and private rates of dental services.

The most favoured areas in France in terms of the number of dental professionals were Paris and its metropolitan area and the departments of the French periphery. In contrast, the departments of Cantal, Creuse, Eure, Haute-Saone, Haute-Loire, Guadeloupe, Indre, Manche, Mayenne, Lozere, Somme, Orne, Yonne, and some islands (eg, Mayotte and Guyane) had the lowest number of dental professionals. Higher numbers of dental schools and greater per capita income were statistically associated with the rates of all analysed dental professionals (general dentists, orthodontists, and oral surgeons) in France. Differences in the distributions found amongst the areas with dental schools in France suggest that this could be an important factor to address so as to enhance the harmonisation in the number of European dental professionals.

Germany showed a different internal pattern of dental professionals than France, with no clearly defined geographic gradient, which could be considered a more efficient distribution. The most homogeneous presence and the highest number of dental faculties are in Germany, which could explain this different pattern of professionals’ distribution. However, many other factors should be analysed. *Rdent, Rorth,* and *RoralSur* were above the 95th percentile in Berlin, Hamburg, and Bädem-Württemberg. Administrative functions (Berlin) and economic reasons (Hamburg is the Länder with the highest per capita income in Germany) might explain this, but this hypothesis was only partially tested in this study and was inconclusive. In contrast, the ratios were below the 5th percentile in Saarland and Sachsen-Anhalt.

The cluster analysis identified 2 main clusters. The most homogeneous group included almost all of the German and French areas above the 95th percentile, suggesting a common trait amongst various dental professionals. Inside this cluster, we found a small subcluster, which included Paris, Hamburg, Alpes-Maritimes, and Berlin. The other large cluster, which was more heterogeneous, only comprised French departments. Thus, Germany grouped all its areas into a single cluster, whilst France divided them between 2 clusters. This finding suggests a higher homogeneity in the distribution of the analysed dental professionals in Germany than that in France.

## Conclusions

Ratios of general dentists and dental specialists to the total population and proportions of dental specialists to general dentists were diverse in the EEA and the UK, and their maximum values were 2.5, 5.7, and 4.1 times the minimum values in the countries analysed, respectively.

Such differences were even found within the same country, as is the case in France and, to a lesser extent, in Germany. Therefore, additional countries and cofactors other than the presence of dental schools and per capita income should be analysed to establish the origin of these differences.

Some suggestions have been proposed to improve dental professional harmonisation on a European level and to solve the lack of uniformity that would be expected in such a developed community.

## Conflict of interest

None disclosed.

## Appendix Supplementary materials

Image, application 1

Image, application 2

Image, application 3

Image, application 4

## Footnotes

Supplementary material associated with this article can be found in the online version at doi:10.1016/j.identj.2023.12.004.
