# Supplementary material for: Differences in the Ratios of General and Dental Specialists in Europe
Source: Int Dent J. 2024 Jan 16;74(3):519–25. doi: 10.1016/j.identj.2023.12.004 (PMC11123524; doi:10.1016/j.identj.2023.12.004)
Supplement: Supplementary file 1 [file mmc1.docx]

**Supplementary Figure 1. Regional distribution of dentists (upper part) and orthodontists (lower part) per 100,000 population in France**. In general, it seems that Paris and its metropolitan area and the regions of the French periphery have higher *Rdent* and *Rorth* values. This difference is much more pronounced in the regions of southern France (Alpes-Maritimes, Bas-Rhin, Bouches-du-Rhone, Gironde, Hérault, Haute-Garonne or Pyrynees-Atlantiques).

(Symbols indicate the presence of Faculty of Odontology)

*
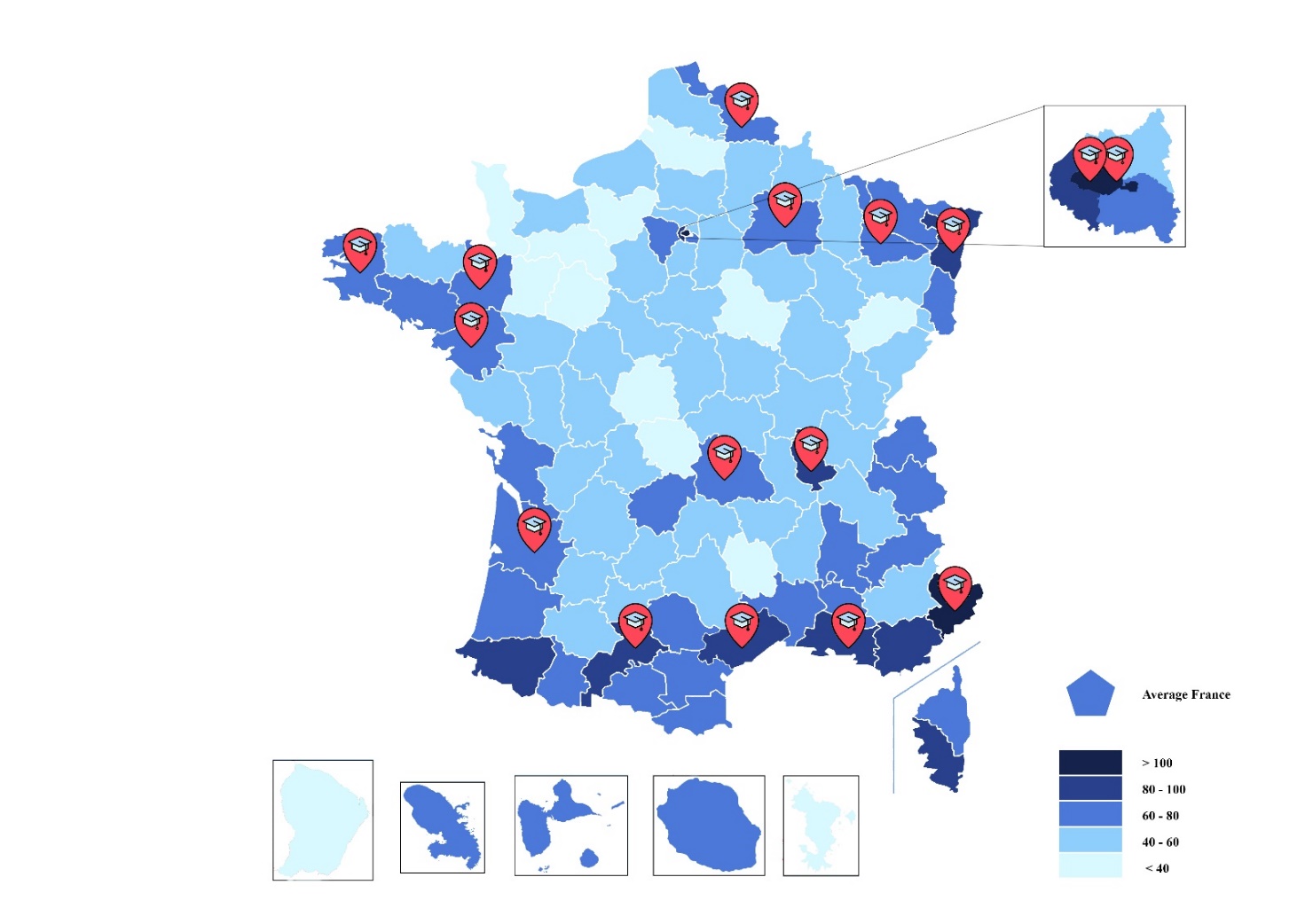
*

*
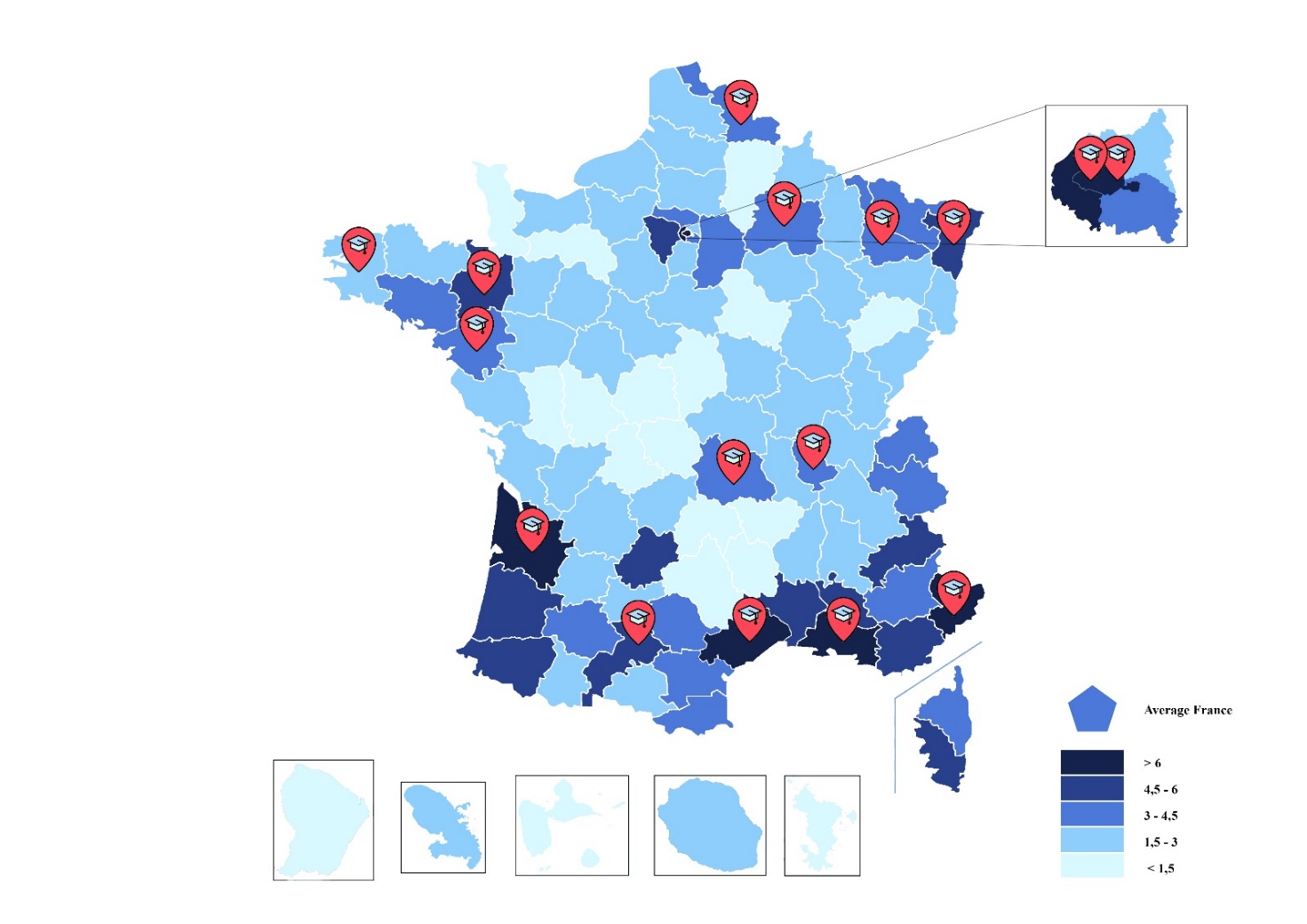
*
