# Supplementary material for: Differences in the Ratios of General and Dental Specialists in Europe
Source: Int Dent J. 2024 Jan 16;74(3):519–25. doi: 10.1016/j.identj.2023.12.004 (PMC11123524; doi:10.1016/j.identj.2023.12.004)

**Supplementary Figure 2. Regional distribution of dentists (upper part) and orthodontists (lower part) per 100,000 population in Germany.** No clearly defined geographical gradient is observed. (Symbols indicate the presence of Faculty of Odontology)


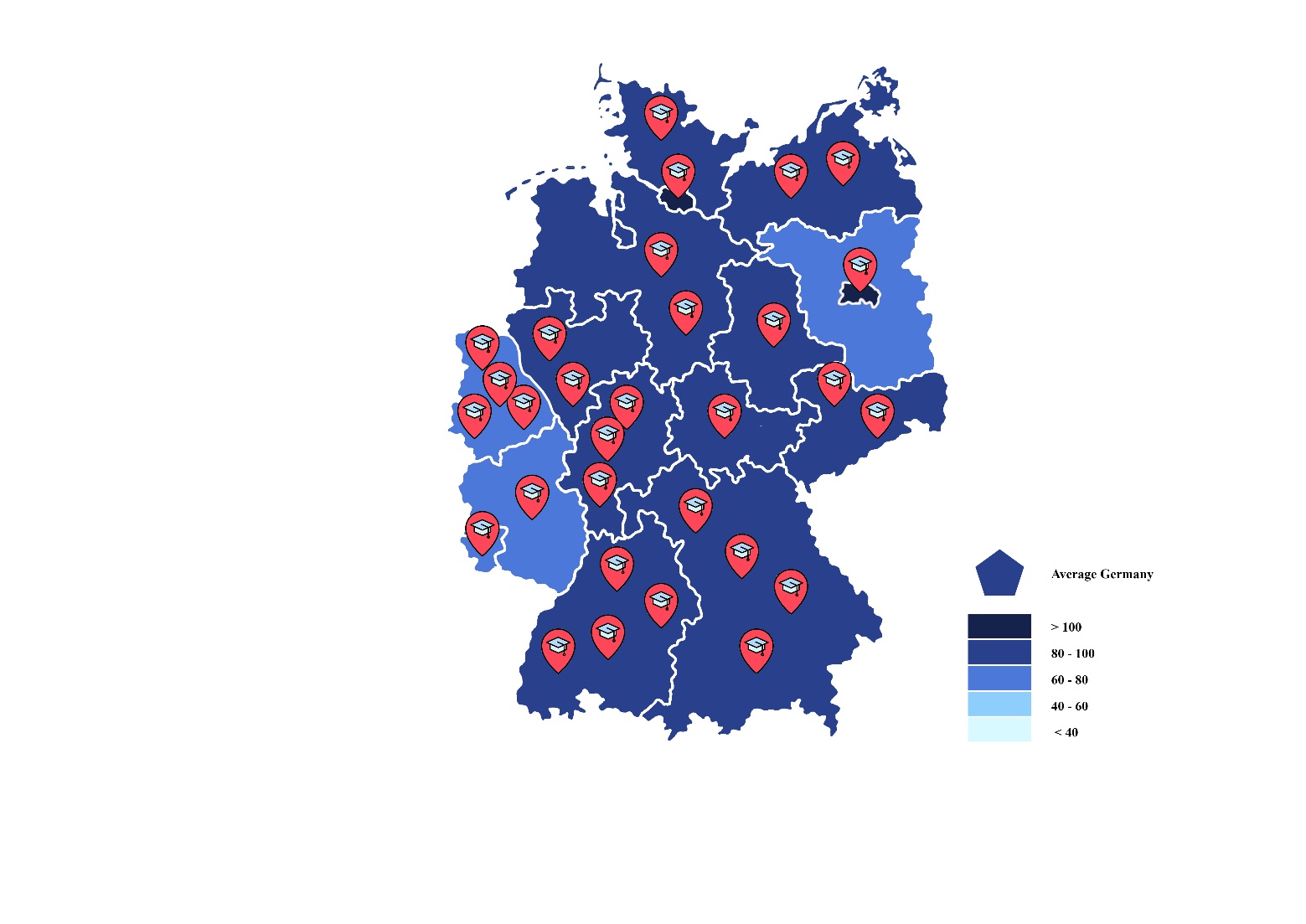

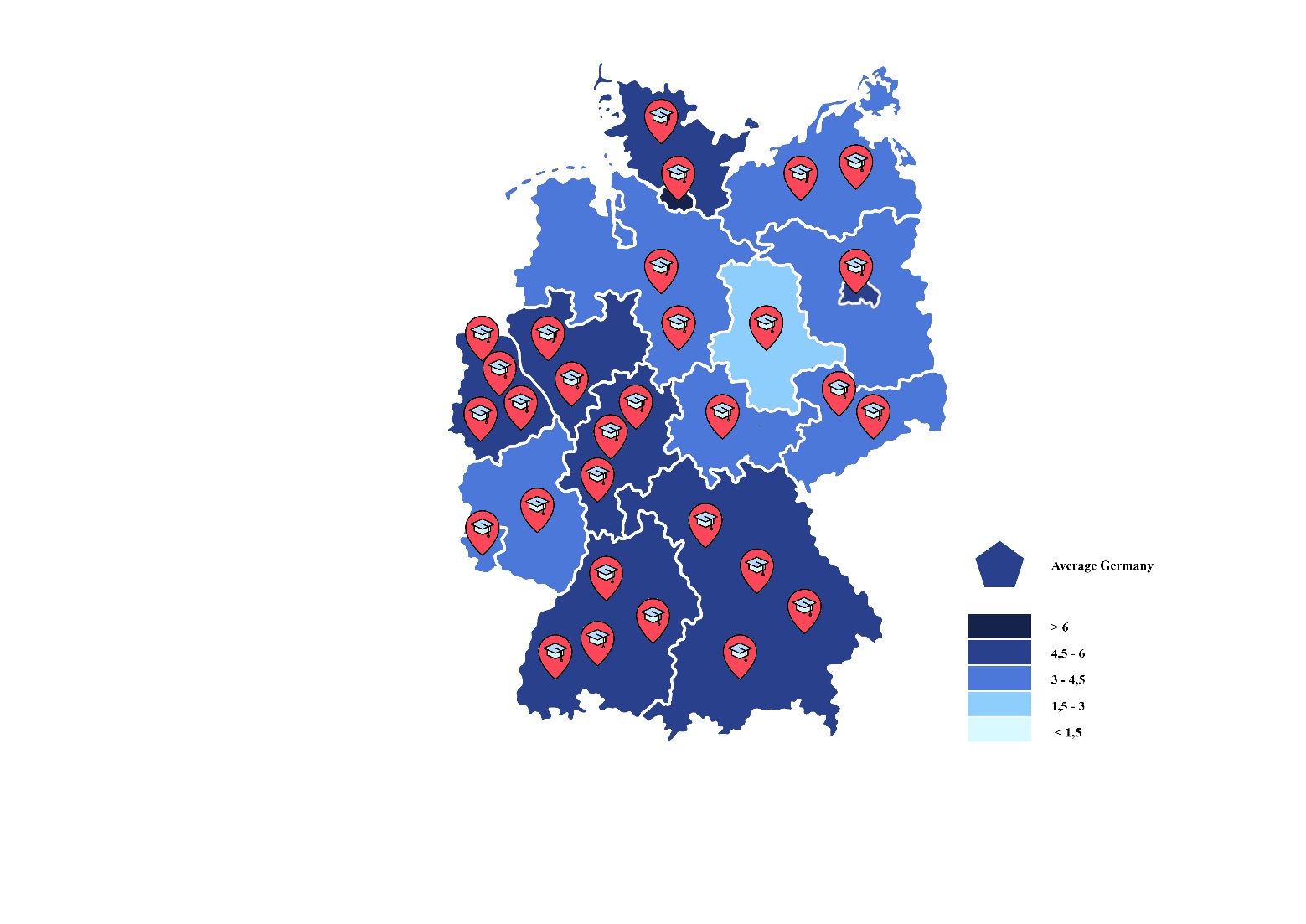

Supplement: Supplementary file 2 [file mmc2.docx]
