# Supplementary material for: Differences in the Ratios of General and Dental Specialists in Europe
Source: Int Dent J. 2024 Jan 16;74(3):519–25. doi: 10.1016/j.identj.2023.12.004 (PMC11123524; doi:10.1016/j.identj.2023.12.004)
Supplement: Supplementary file 3 [file mmc3.docx]

Supplementary Table 1. Regional distribution of dentists and specialists in France

| *Region* | *pop* | *dent* | *Rdent* | *Orth (%)* | *Rorth* | *oralSur* | *RoralSur* |
| --- | --- | --- | --- | --- | --- | --- | --- |
| Ain | 665,391 | 352 | 52.9 | 18 (5.1%) | 2.7 | 0 | 0 |
| Aisne | 524,403 | 238 | 45.4 | 5 (2.1%) | 1.0 | 0 | 0 |
| Allier | 331,757 | 172 | 51.8 | 6 (3.5%) | 1.8 | 0 | 0 |
| Alpes-de-Haute-Provence | 165,582 | 99 | 59.8 | 7 (7.1%) | 4.2 | 0 | 0 |
| Hautes-Alpes | 141,059 | 99 | 70.2 | 7 (7.1%) | 5.0 | 1 | 0.7 |
| Alpes-Maritimes | 1,103,555 | 1,331 | 120.6 | 80 (6.0%) | 7.2 | 2 | 0.2 |
| Ardèche | 330,865 | 165 | 49.9 | 8 (4.8%) | 2.4 | 0 | 0 |
| Ardennes | 265,285 | 119 | 44.9 | 5 (4.2%) | 1.9 | 0 | 0 |
| Ariege | 153,126 | 94 | 61.4 | 3 (3.2%) | 2.0 | 1 | 0.7 |
| Aube | 311,083 | 154 | 49.5 | 8 (5.2%) | 2.6 | 0 | 0 |
| Aude | 379,844 | 235 | 61.9 | 14 (6.0%) | 3.7 | 1 | 0.3 |
| Aveyron | 280,254 | 161 | 57.4 | 4 (2.5%) | 1.4 | 0 | 0 |
| Bouches-du-Rhône | 2,062,499 | 1,927 | 93.4 | 127 (6.6%) | 6.2 | 8 | 0.4 |
| Calvados | 695,310 | 364 | 52.4 | 20 (5.5%) | 2.9 | 2 | 0.3 |
| Cantal | 143,280 | 71 | 49.6 | 1 (1.4%) | 0.7 | 0 | 0 |
| Charente | 350,298 | 162 | 46.2 | 10 (6.2%) | 2.9 | 2 | 0.6 |
| Charente-Maritime | 659,482 | 417 | 63.2 | 18 (4.3%) | 2.7 | 2 | 0.3 |
| Cher | 296,904 | 135 | 45.5 | 3 (2.2%) | 1.0 | 0 | 0 |
| Corrèze | 238,445 | 143 | 60.0 | 7 (4.9%) | 2.9 | 1 | 0.4 |
| Corse-du-Sud | 162,314 | 130 | 80.1 | 8 (6.2%) | 4.9 | 1 | 0.6 |
| Haute-Corse | 187,151 | 116 | 62.0 | 6 (5.2%) | 3.2 | 0 | 0 |
| Côte-d’Or | 534,424 | 308 | 57.6 | 8 (2.6%) | 1.5 | 3 | 0.6 |
| Côtes-d’Armor | 601,354 | 342 | 56.9 | 16 (4.7%) | 2.7 | 7 | 1.2 |
| Creuse | 113,711 | 43 | 37.8 | 1 (2.3%) | 0.9 | 0 | 0 |
| Dordogne | 411,087 | 205 | 49.9 | 8 (3.9%) | 1.9 | 2 | 0.5 |
| Doubs | 547,572 | 306 | 55.9 | 14 (4.6%) | 2.6 | 0 | 0 |
| Drôme | 524,506 | 319 | 60.8 | 10 (3.1%) | 1.9 | 0 | 0 |
| Eure | 593,885 | 215 | 36.2 | 9 (4.2%) | 1.5 | 0 | 0 |
| Eure-et-Loir | 427,776 | 172 | 40.2 | 9 (5.2%) | 2.1 | 0 | 0 |
| Finistère | 922,797 | 609 | 66.0 | 20 (3.3%) | 2.2 | 3 | 0.3 |
| Gard | 753,436 | 535 | 71.0 | 35 (6.5%) | 4.6 | 4 | 0.5 |
| Haute-Garonne | 1,452,055 | 1,368 | 94.2 | 76 (5.6%) | 5.2 | 14 | 1.0 |
| Gers | 192,279 | 107 | 55.6 | 6 (5.6%) | 3.1 | 0 | 0 |
| Gironde | 1,681,330 | 1,342 | 79.8 | 108 (8.0%) | 6.4 | 8 | 0.5 |
| Hérault | 1,217,787 | 1,056 | 86.7 | 90 (8.5%) | 7.4 | 9 | 0.7 |
| Ille-et-Villaine | 1,107,860 | 758 | 68.4 | 53 (7.0%) | 4.8 | 8 | 0.7 |
| Indre | 215,031 | 80 | 37.2 | 3 (3.8%) | 1.4 | 0 | 0 |
| Indre-et-Loire | 613,406 | 308 | 50.2 | 13 (4.2%) | 2.1 | 1 | 0.2 |
| Isère | 1,285,915 | 739 | 57.5 | 34 (4.6%) | 2.6 | 1 | 0.1 |
| Jura | 257,128 | 126 | 49.0 | 4 (3.2%) | 1.6 | 0 | 0 |
| Landes | 422,332 | 286 | 67.7 | 19 (6.6%) | 4.5 | 0 | 0 |
| Loir-et-Cher | 325,598 | 159 | 48.8 | 8 (5.0%) | 2.5 | 0 | 0 |
| Loire | 767,549 | 425 | 55.4 | 18 (4.2%) | 2.3 | 2 | 0.3 |
| Haute-Loire | 227,224 | 114 | 50.2 | 2 (1.8%) | 0.9 | 0 | 0 |
| Loire-Atlantique | 1,478,101 | 1,005 | 68.0 | 64 (6.4%) | 4.3 | 5 | 0.3 |
| Loiret | 686,200 | 318 | 46.3 | 15 (4.7%) | 2.2 | 0 | 0 |
| Lot | 174,522 | 100 | 57.3 | 8 (8.0%) | 4.6 | 0 | 0 |
| Lot-et-Garonne | 329,135 | 165 | 50.1 | 6 (3.6%) | 1.8 | 0 | 0 |
| Lozère | 76,573 | 30 | 39.2 | 1 (3.3%) | 1.3 | 0 | 0 |
| Maine-et-Loire | 825,241 | 398 | 48.2 | 22 (5.5%) | 2.7 | 3 | 0.4 |
| Manche | 491,281 | 191 | 38.9 | 7 (3.7%) | 1.4 | 0 | 0 |
| Marne | 562,545 | 403 | 71.6 | 23 (5.7%) | 4.1 | 4 | 0.7 |
| Haute-Marne | 166,343 | 76 | 45.7 | 4 (5.3%) | 2.4 | 0 | 0 |
| Mayenne | 305,870 | 110 | 36.0 | 6 (5.5%) | 2.0 | 2 | 0.7 |
| Meurthe-et-Moselle | 731,006 | 545 | 74.6 | 22 (4.0%) | 3.0 | 5 | 0.7 |
| Meuse | 178,156 | 76 | 42.7 | 4 (5.3%) | 2.2 | 0 | 0 |
| Morbihan | 770,921 | 502 | 65.1 | 32 (6.4%) | 4.2 | 6 | 0.8 |
| Moselle | 1,044,398 | 672 | 64.3 | 37 (5.5%) | 3.6 | 5 | 0.5 |
| Nièvre | 199,373 | 97 | 48.7 | 3 (3.1%) | 1.5 | 0 | 0 |
| Nord | 2,606,873 | 1,594 | 61.1 | 109 (6.8%) | 4.2 | 4 | 0.2 |
| Oise | 833,013 | 342 | 41.1 | 17 (5.0%) | 2.0 | 0 | 0 |
| Orne | 273,214 | 106 | 38.8 | 2 (1.9%) | 0.7 | 0 | 0 |
| Pas-de-Calais | 1,456,555 | 708 | 48.6 | 39 (5.5%) | 2.7 | 2 | 0.1 |
| Puy-de-Dôme | 672,494 | 501 | 74.5 | 24 (4.8%) | 3.6 | 3 | 0.4 |
| Pyrénées- Atlantiques | 691,770 | 611 | 88.3 | 35 (5.7%) | 5.1 | 3 | 0.4 |
| Hautes-Pyrénées | 231,014 | 179 | 77.5 | 6 (3.4%) | 2.6 | 0 | 0 |
| Pyrénées-Orientales | 485,396 | 363 | 74.8 | 16 (4.4%) | 3.3 | 2 | 0.4 |
| Bas-Rhin | 1,156,546 | 1,060 | 91.7 | 62 (5.8%) | 5.4 | 4 | 0.3 |
| Haut-Rhin | 768,557 | 498 | 64.8 | 20 (4.0%) | 2.6 | 0 | 0 |
| Rhône | 1,912,073 | 1,534 | 80.2 | 84 (5.5%) | 4.4 | 8 | 0.4 |
| Haute-Saône | 232,531 | 85 | 36.6 | 0 | 0 | 0 | 0 |
| Saône-et-Loire | 546,577 | 267 | 48.8 | 8 (3.0%) | 1.5 | 0 | 0 |
| Sarthe | 564,588 | 224 | 39.7 | 12 (5.4%) | 2.2 | 0 | 0 |
| Savoie | 442,054 | 332 | 75.1 | 17 (5.1%) | 3.8 | 1 | 0.2 |
| Haute-Savoie | 850,125 | 612 | 72.0 | 37 (6.0%) | 4.4 | 0 | 0 |
| Paris | 2,139,907 | 3,297 | 154.1 | 166 (5.0%) | 7.8 | 31 | 1.4 |
| Seine-Maritime | 1,253,596 | 605 | 48.3 | 27 (4.5%) | 2.2 | 2 | 0.2 |
| Seine-et-Marne | 1,443,796 | 742 | 51.4 | 44 (5.9%) | 3.0 | 0 | 0 |
| Yvelines | 1,463,709 | 903 | 61.7 | 76 (8.4%) | 5.2 | 3 | 0.2 |
| Deux-Sèvres | 374,426 | 156 | 41.7 | 5 (3.2%) | 1.3 | 0 | 0 |
| Somme | 566,328 | 201 | 35.5 | 10 (5.0%) | 1.8 | 0 | 0 |
| Tarn | 393,046 | 251 | 63.9 | 13 (5.2%) | 3.3 | 0 | 0 |
| Tarn-et-Garonne | 264,216 | 141 | 53.4 | 7 (5.0%) | 2.6 | 0 | 0 |
| Var | 1,095,564 | 889 | 81.1 | 51 (5.7%) | 4.7 | 2 | 0.2 |
| Vaucluse | 562,928 | 402 | 71.4 | 32 (8.0%) | 5.7 | 0 | 0 |
| Vendée | 699,296 | 376 | 53.8 | 18 (4.8%) | 2.6 | 3 | 0.4 |
| Vienne | 440,164 | 188 | 42.7 | 6 (3.2%) | 1.4 | 0 | 0 |
| Haute-Vienne | 369,805 | 190 | 51.4 | 5 (2.6%) | 1.4 | 0 | 0 |
| Vosges | 358,175 | 207 | 57.8 | 7 (3.4%) | 2.0 | 4 | 1.1 |
| Yonne | 330,074 | 116 | 35.1 | 3 (2.6%) | 0.9 | 0 | 0 |
| Territoire de Belfort | 137,714 | 76 | 55.2 | 3 (3.9%) | 2.2 | 1 | 0.7 |
| Essonne | 1,315,404 | 702 | 53.4 | 38 (5.4%) | 2.9 | 2 | 0.2 |
| Hauts-de-Seine | 1,643,080 | 1,522 | 92.6 | 114 (7.5%) | 6.9 | 9 | 0.5 |
| Seine-Saint-Denis | 1,678,367 | 882 | 52.6 | 38 (4.3%) | 2.3 | 3 | 0.2 |
| Val-de-Marne | 1,434,351 | 993 | 69.2 | 52 (5.3%) | 3.6 | 10 | 0.7 |
| Val-d’Oise | 1,276,534 | 714 | 55.9 | 41 (5.7%) | 3.2 | 1 | 0.1 |
| Guadeloupe | 372,939 | 263 | 70.5 | 4 (1.5%) | 1.1 | 0 | 0 |
| Martinique | 350,373 | 243 | 69.4 | 8 (3.3%) | 2.3 | 1 | 0.3 |
| Guyane | 294,436 | 86 | 29.2 | 1 (1.2%) | 0.3 | 0 | 0 |
| La Réunion | 868,846 | 586 | 67.4 | 22 (3.8%) | 2.5 | 0 | 0 |
| Mayotte | 299,348 | 28 | 9.4 | 0 | 0 | 0 | 0 |
| FRANCE | 65,627,454 | 43,839 | 66.8 | 2427 (5.5%) | 3.7 | 212 (0.5%) | 0.3 |
