# Supplementary material for: Differences in the Ratios of General and Dental Specialists in Europe
Source: Int Dent J. 2024 Jan 16;74(3):519–25. doi: 10.1016/j.identj.2023.12.004 (PMC11123524; doi:10.1016/j.identj.2023.12.004)
Supplement: Supplementary file 4 [file mmc4.docx]

**Supplementary Table 2. Regional distribution of dentists and specialists in Germany**

| *Region* | *pop* | *dent* | *Rdent.* | *orth (%)* | *Rorth* | *oralSur* | *RoralSur* |
| --- | --- | --- | --- | --- | --- | --- | --- |
| Baden-Württemberg | 10,924,475 | 9,284 | 85.0 | 556 (6.0%) | 5.1 | 586 | 5.4 |
| Bayern | 12,896,719 | 12,063 | 93.5 | 634 (5.3%) | 4.9 | 552 | 4.3 |
| Berlin | 3,549,836 | 4,330 | 122.0 | 196 (4.5%) | 5.5 | 157 | 4.4 |
| Brandenburg | 2,491,196 | 1,943 | 78.0 | 91 (4.7%) | 3.7 | 62 | 2.5 |
| Bremen | 675,908 | 550 | 81.4 | 22 (4.0%) | 3.3 | 33 | 4.9 |
| Hamburg | 1,797,346 | 2,074 | 115.4 | 112 (5.4%) | 6.2 | 89 | 5.0 |
| Hessen | 6,172,808 | 5,666 | 91.8 | 353 (6.2%) | 5.7 | 320 | 5.2 |
| Mecklenburg-Vorpommern | 1,611,628 | 1,395 | 86.6 | 61 (4.4%) | 3.8 | 56 | 3.5 |
| Niedersachsen | 7,959,369 | 6,529 | 82.0 | 302 (4.6%) | 3.8 | 239 | 3.0 |
| Nordrhein | 9,660,708 | 8,368 | 86.6 | 440 (5.3%) | 4.6 | 437 | 4.5 |
| Rheinland-Pfalz | 4,056,783 | 3,142 | 77.4 | 129 (4.1%) | 3.2 | 157 | 3.9 |
| Saarland | 998,045 | 694 | 69.5 | 34 (4.9%) | 3.4 | 52 | 5.2 |
| Sachsen | 4,071,473 | 3,853 | 94.6 | 169 (4.4%) | 4.2 | 151 | 3.7 |
| Sachsen-Anhalt | 2,240,338 | 1,827 | 81.6 | 66 (3.6%) | 2.9 | 37 | 1.7 |
| Schleswig-Holstein | 2,879,453 | 2,403 | 83.4 | 133 (5.5%) | 4.6 | 134 | 4.7 |
| Thüringen | 2,157,739 | 1,943 | 90.0 | 81 (4.2%) | 3.8 | 56 | 2.6 |
| Westfalen-Lippe | 8,249,950 | 6,527 | 79.1 | 373 (5.7%) | 4.5 | 334 | 4.0 |
| Bundesgebiet (Total) | 82,358,329 | 72,591 | 88.1 | 3752 (5.2%) | 4.6 | 3452 | 4.2 |
